# Humanised Mice in Cutaneous Leishmaniasis—T‐Cell Recruitment Into Human Skin Transplants After *Leishmania major* Infection

**DOI:** 10.1111/exd.70131

**Published:** 2025-07-01

**Authors:** Ling Miao, Henning Klapproth, Michael R. Stepkes, Joanna Wegner, Esther von Stebut

**Affiliations:** ^1^ Department of Dermatology, Faculty of Medicine University of Cologne Cologne Germany; ^2^ Department of Dermatology Johannes Gutenberg University Mainz Germany

## Abstract

Treatment against leishmaniasis is associated with severe side effects, high costs, and parasitic resistance. Preclinical models such as humanised mice would aid therapeutic improvement or the development of a vaccine. We developed a model in which human skin transplants on immunodeficient mice are infected with *Leishmania major* . Parasite inoculation of the skin transplant led to a robust infection with increasing numbers of parasites in the skin and visceral organs. In addition, intraperitoneally co‐administered allogeneic peripheral blood mononuclear cells (PBMCs) were strongly recruited to skin lesions, with ≥ 65% of the cells being positive for anti‐human CD45; we identified ~20% CD4^+^ and ~50% CD8^+^ human T cells. The number of skin‐resident macrophages or dendritic cells was unaltered compared to healthy skin prior to transplantation, and PBMC administration did not alter their numbers. Together, we show that parasitic infection provides a strong inflammatory signal that leads to recruitment of T cells into skin transplants. The presence of antigen‐presenting cells in the transplants—as an important prerequisite for proper APC‐T‐cell interaction—recreates a fully human skin microenvironment that allows for stroma/immune cell interactions upon infection. This model may be of high interest to researchers interested in translating skin research questions into the human system in vivo.

## 1 Background

Mice xenotransplanted with human cells (so‐called “humanised mice”) are often utilised to gain insights into human‐specific pathophysiology. As preclinical models, they can serve to bridge the gap between mechanisms discovered in experimental models and human disease. Utilised mouse strains are generally immunodeficient but harbour human genes, cells, tissues or organs that were transferred. They are deployed to, e.g., test novel therapies or to assess if molecular mechanisms in humans are similar to those discovered in mice.

Leishmaniasis is a parasitic infection transmitted by infected sand flies. Disease presentation ranges from cutaneous, mucocutaneous to visceral forms. Two aspects determine disease outcome: the parasite subspecies and—very importantly—the host immune status [1]. Treatment against leishmaniasis consists of anti‐parasitic drugs that are costly and have numerous, in part, severe side effects, and treatment is often not available in endemic countries. A vaccine is not available yet. The establishment of a humanised mouse model for cutaneous leishmaniasis would aid the development of novel therapeutic options, both topically and systemically, and/or a vaccine.

## 2 Question Addressed

Previously, we have assessed the suitability of humanised mice that were T‐, B‐ and NK cell‐deficient and adoptively transferred with human peripheral blood mononuclear cells (PBMC). *L. major* infection led to parasite replication in lesions and weak immigration of human T cells into the infection site [2]. In addition, human IFN was barely detectable, indicating an inability of murine antigen‐presenting cells (APCs) to present antigen across species. We have now tested a similar approach utilising a more physiological situation in which immune cell interaction with human stroma is possible and assessed as a first step if immune cell recruitment to skin lesions can be achieved.

## 3 Experimental Design

**FIGURE 1:**
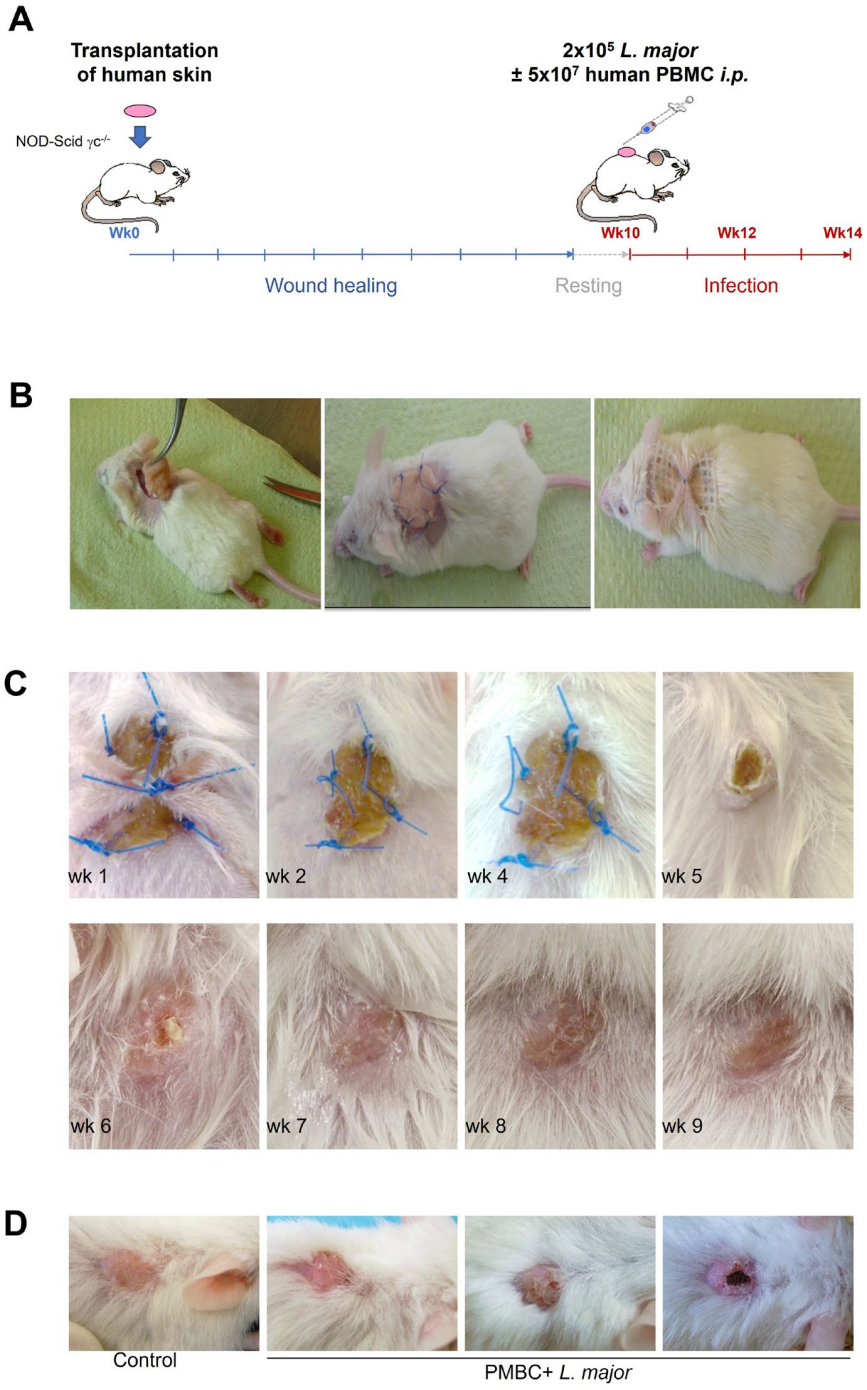
Human skin transplantation onto immunodeficient mice. Transplantation of 2 × 1 cm tissue specimen onto backs of NOD‐Scidγc^−/−^ (NSG) mice deficient for T, B and NK cells. After completion of wound healing and full recovery followed by some weeks of rest, mice were infected with 2 × 10^5^ metacyclic promastigotes of *Leishmania major* . Simultaneously, human PBMCs were prepared from buffy coats, and 5 × 10^7^ PBMC/100 μL were injected intraperitoneally. (A) Schematic representation of experimental procedures. (B) Procedure of skin removal, placement of human skin into the defect, suture and gauze placement completed by creation of a skin pouch for fixation. Pouches spontaneously resolved within a maximum of 3 days. (C) Close up presentation of wound healing for 9 weeks post‐transplantation. Animal housing and all the experimental procedures were authorised by the Animal Care and Use Committees of Rhineland Palatinate. Ethical approval and informed consent were obtained from donors of excess skin. (D) Representative figures of skin lesions after infection in week 14 post‐transplantation.

Groups of T‐, B‐ and NK cell‐deficient NOD‐Scidɣc^−/−^ (NSG) mice were transplanted with 1 × 2 cm of human skin onto their backs (Figure 1A–D). Skin was obtained from excess material from plastic or reconstructive surgery after obtaining informed consent from the donor. After healing for ~2–3 months, mice were infected intradermally with 2 × 10^5^ infectious‐stage metacyclic parasites together with intraperitoneal administration of human, allogeneic PBMC from healthy donors. After several weeks, lesional parasite burdens were assessed. In addition, two weeks post‐infection, using immunohistochemistry, skin lesions were assessed for the presence of human inflammatory cells using antibodies against CD45 (myeloid cells, Dako M0701), CD3 (T cells, Leica), CD4 (Th cells, Cell Marque 104R‐25), CD8 (cytotoxic T cells, Dako M7103), CD1a (dendritic cells, Dako, m3571) and CD68 (MΦ, Dako, M0876), all of which showed no murine cross‐reactivity.

## 4 Results

**FIGURE 2:**
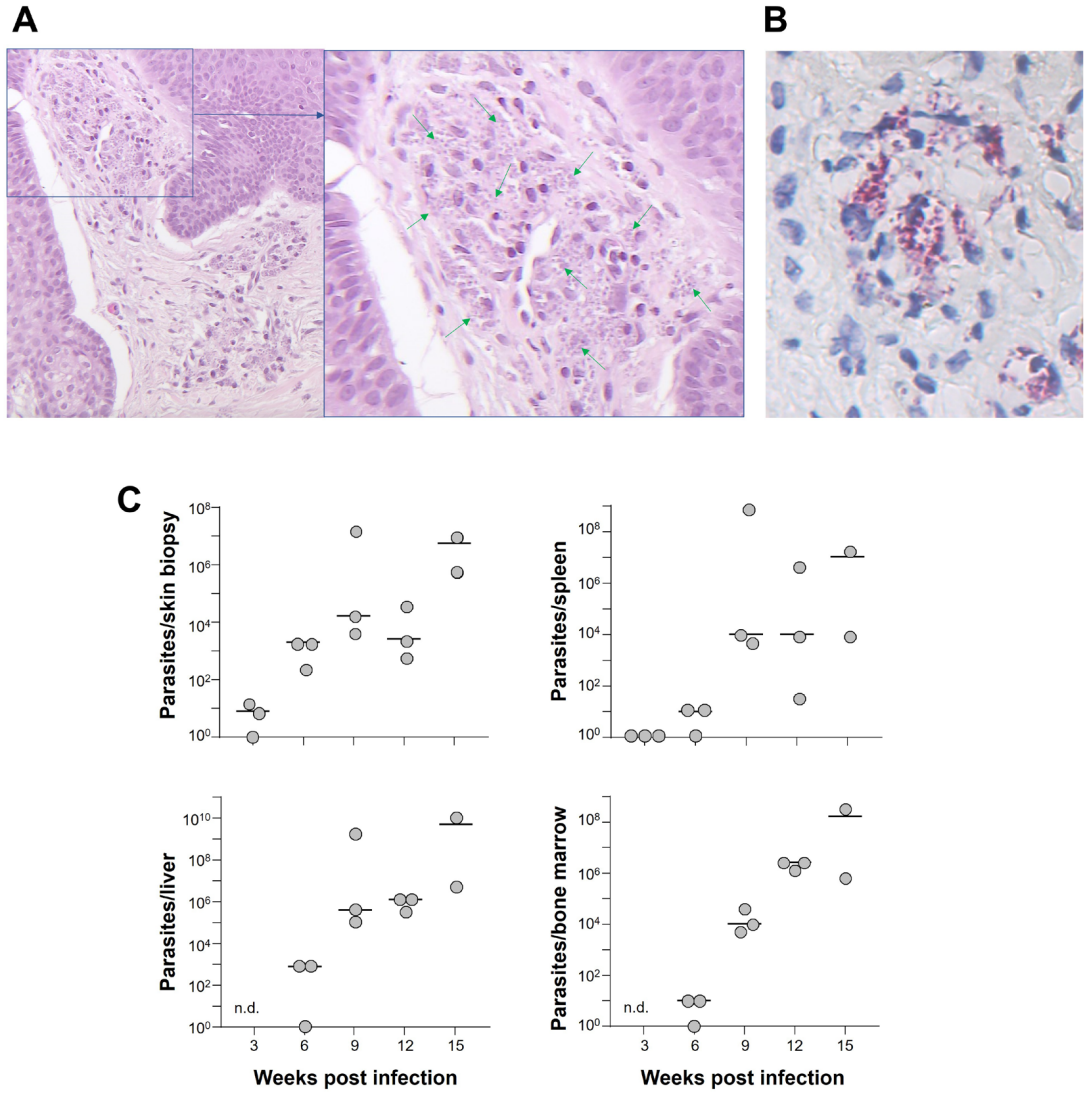
Experimental infection of skin transplant with *Leishmania major*. Skin transplants were infected with 2 × 10^5^ *L. major* parasites. (A) Skin tissue was biopsied in week 2, and parasites were detected in histiocytes by Giemsa staining to confirm infection. (B) Anti‐CD1a (clone MTB1) staining to visualise *Leishmania* amastigotes. (C) At the indicated time points, mice were sacrificed, and parasite burdens in skin, spleen, liver and bone marrow were determined using limiting dilution assays. Circles represent individual parasite numbers, and bars indicate means.

**FIGURE 3:**
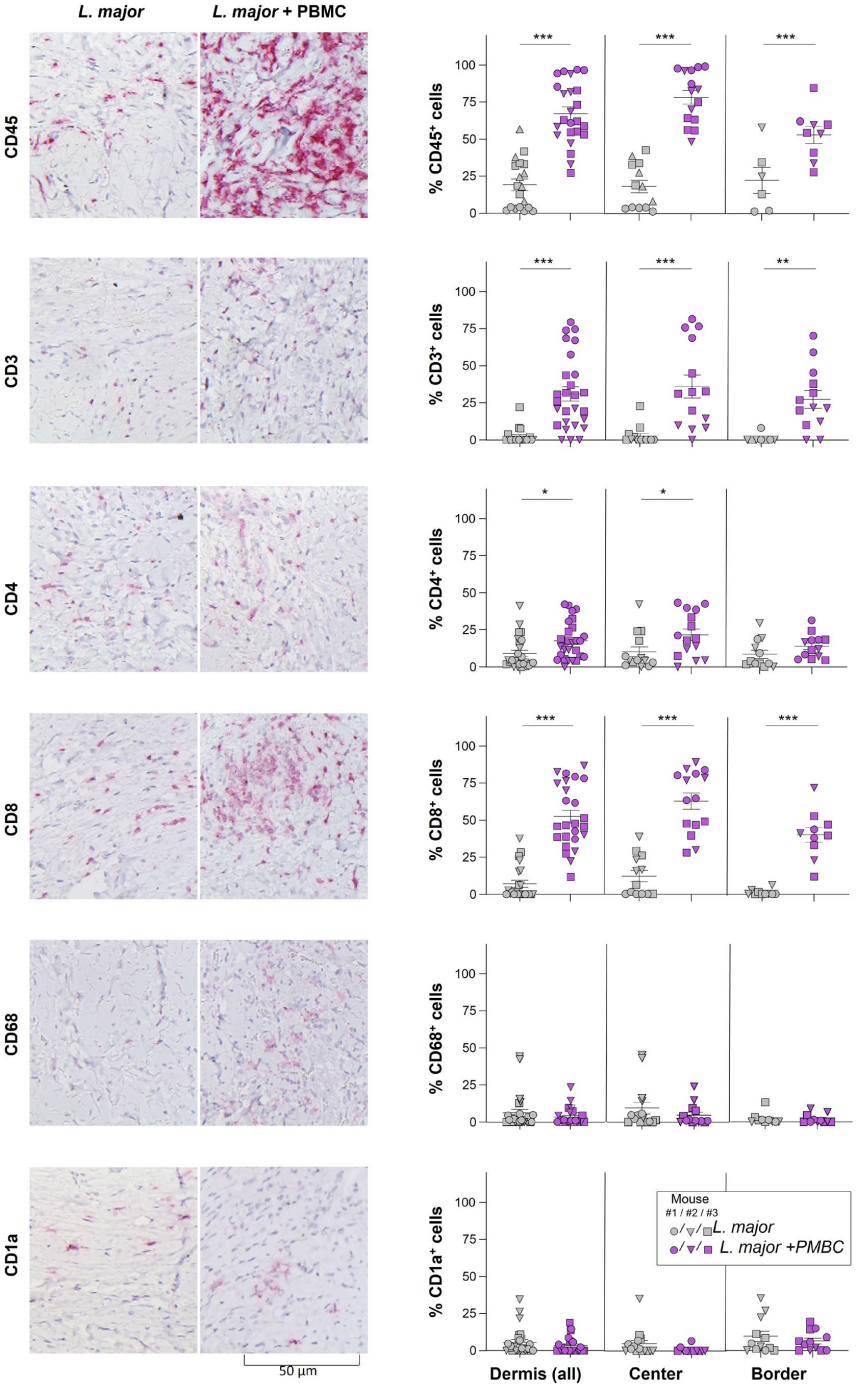
After intraperitoneal co‐administration of PBMC, human T cells are recruited to *Leishmania major*‐infected human skin transplants. NOD‐Scidγc^−/−^ (NSG) mice received human skin transplants that were infected with *L. major* after 14 weeks (4 weeks post‐healing), while receiving 2 × 10^7^ allogeneic PBMC intraperitoneally. Control mice did not receive PBMC. After 2 weeks, skin was biopsied, and the inflammatory infiltrate was characterised by immunohistochemistry using antibodies specific for human CD45 (myeloid cells), CD3, CD4, CD8 (T cell markers), CD68 (macrophages) or CD1a (dendritic cells); cross‐reactivity with murine tissue was excluded prior to staining. The frequency of cells positive for each marker compared to all cells was determined (3–5 representative pictures of each mouse, *n* = 3 independent mice) using light microscopy (200 ×) and Qpath software, version 0.5.0. Data are shown as individual measurements; bars represent means. Statistical differences were calculated using Graphpad software with *p ≤ 0.05, **p ≤ 0.01 and ***p ≤ 0.005.

First, transplanted mice were assessed for lesion development over the course of several weeks. Erythema was observed, papules or plaques were not always seen nor was ulceration (Figure 3). To assess if parasite inoculation established a skin infection, we obtained biopsies of infected tissue (Figure 2A). Histology revealed a strong inflammatory infiltrate in the upper papillary dermis containing numerous histiocytes harbouring amastigotes of *L. major* . Only a few lymphocytes were detected. Visualisation of *L. major* parasites by staining with anti‐CD1a revealed that the majority resided intracellularly (Figures 2B).

In addition, three mice were sacrificed each week between weeks 3 and 15 post‐infection, and the parasite burden of skin, spleens, liver and bone marrow was determined using limited dilution assays (Figure 2C) [3]. We observed a steady increase of parasite numbers in all tissues over the course of 3 months, reaching the highest levels in week 15 post‐infection. These data indicate that despite a lack of papules in lesions, the infection with *L. major* establishes itself over the course of the next weeks, and parasites also invade internal organs and the bone marrow, as would be seen in immunodeficient hosts and/or visceral leishmaniasis.

Finally, we assessed if i.p. administration of human allogeneic PBMC led to recruitment of inflammatory cells into lesions. Lesional tissue was harvested after 2 weeks, and the inflammatory infiltrate was characterised using antibodies specific for human markers (Figure 3). First, upon administration of PBMC, a dramatic increase in the frequency of hCD45^+^ cells in infected skin was detected (*p* ≤ 0.002), the majority of which were CD3^+^ T cells. The highest number of cells was found in the centre of the granuloma. In line with previous findings [2], as a control, we also injected PBMC without concomitant infection, which did not recruit PMBC to skin transplants (data not shown). Staining for CD4 and CD8 revealed that both T helper cells and CD8^+^ T cells were recruited from the peritoneum to the skin upon infection; interestingly, the number of cytotoxic T cells was higher in lesional tissue as compared to CD4^+^ Th cells. The number of skin‐resident macrophages (MΦ, CD68^+^) as well as dendritic cells (DC, both dermal and epidermal, CD1a^+^) was unaltered upon PBMC administration.

## 5 Conclusions and Perspectives

In summary, our data indicate that a stimulus such as *L. major* infection provides a strong inflammatory signal that leads to recruitment of adoptively transferred T cells (i.p.) into previously transplanted human skin. Interestingly, and as observed before, the frequency of CD1a^+^ epidermal Langerhans cells as well as dermal DC and MΦ was not altered compared to the numbers in the skin before transplantation, highlighting their long‐lived nature [4]. In the setting of cutaneous leishmaniasis, this is highly advantageous since the major host cells for the parasites are skin‐resident MΦ. Within MΦ, parasites replicate early on, and at a later stage, MΦ are responsible for parasite elimination after being activated via T cell‐derived IFN [1]. In contrast, skin DCs capture parasite antigen and are responsible for T cell priming [1, 5]. Thus, the presence of both MΦ and DC in human skin 10+ weeks post‐transplantation is an important prerequisite for further experiments requiring functional APC.

Previously, a humanised mouse model for psoriasis was reported using skin‐transplanted mice. In that setting, expansion of co‐transferred skin‐resident T cells upon engraftment of even pre‐psoriatic skin led to the development of psoriatic lesions in transplants within 8 weeks [6]. In our hands, T cell proliferation of skin‐circulating human T cells was not observed, not even after inflammation/infection.

In this study, for these proof‐of‐concept experiments, we have utilised allogenic PBMC with the intention to assess if T cell recruitment to skin can be achieved, aware of limitations regarding APC‐T cell interactions due to HLA mismatch. For future experiments, utilisation of syngeneic PBMC has promising potential for the proper imitation of immune cell interactions in a fully human tissue microenvironment. Alternatively, others have used HLA‐transgenic mice and HLA‐matched donor PBMC in humanisation experiments [7, 8]. An application of HLA‐transgenic mice in infectious diseases has not been reported and still has the disadvantage that murine stroma will interact with human cells.

In summary, by now establishing that human PBMC transferred i.p. are recruited to human skin transplants upon an inflammatory infectious challenge and after confirming that human DC and MΦ (subsets) are still detectable weeks post‐transplant healing, we aim to further improve this model in the future by utilising syngeneic PBMCs, allowing for proper interaction of human cells with the infected tissue of the same donor. All in all, this mouse model recreates a fully human tissue microenvironment, allowing for stroma/immune interactions which may be of high interest for researchers interested in translating their research in skin inflammation or therapeutic interventions. A model such as this one may have the potential to allow for better assessment of the requirements for healing, for therapeutic interventions and for vaccine development in patients.

## Author Contributions

E.S. and M.R.S. designed the research; M.R.S. and J.W. performed the experiments; M.R.S., L.M. and H.K. performed data analysis; E.S. wrote the manuscript. All authors have read and approved the final manuscript.

## Conflicts of Interest

The authors declare no conflicts of interest.

## Data Availability

The data that support the findings of this study are available on request from the corresponding author. The data are not publicly available due to privacy or ethical restrictions.

## References

[1] D. Sacks and N. Noben‐Trauth , “The Immunology of Susceptibility and Resistance to *Leishmania Major* in Mice,” Nature Reviews. Immunology 2 (2002): 845–858. 10.1038/nri933 12415308

[2] M. R. Fischer , A. I. Schermann , T. Twelkmeyer , et al., “Humanized Mice in Cutaneous Leishmaniasis‐Suitability Analysis of Human PBMC Transfer Into Immunodeficient Mice,” Experimental Dermatology 28 (2019): 1087–1090. 31260571 10.1111/exd.13999

[3] E. von Stebut , J. M. Ehrchen , Y. Belkaid , et al., “Interleukin 1alpha Promotes Th1 Differentiation and Inhibits Disease Progression in Leishmania Major‐Susceptible BALB/c Mice,” Journal of Experimental Medicine 198 (2003): 191–199. 12860932 10.1084/jem.20030159 PMC2194079

[4] J. Hemmerling , J. Wegner‐Kops , E. von Stebut , et al., “Human Epidermal Langerhans Cells Replenish Skin Xenografts and Are Depleted by Alloreactive T Cells In Vivo,” Journal of Immunology 187 (2011): 1142–1149. 10.4049/jimmunol.1001491 21697461

[5] E. von Stebut , “Leishmaniasis,” Journal Der Deutschen Dermatologischen Gesellschaft 13, no. 3 (2015): 191–200. 25721626 10.1111/ddg.12595

[6] O. Boyman , H. P. Hefti , C. Conrad , B. J. Nickoloff , M. Suter , and F. O. Nestle , “Spontaneous Development of Psoriasis in a New Animal Model Shows an Essential Role for Resident T Cells and Tumor Necrosis Factor‐Alpha,” Journal of Experimental Medicine 199 (2004): 731–736. 14981113 10.1084/jem.20031482 PMC2213300

[7] D. V. Serreze , M. Niens , J. Kulik , and T. P. DiLorenzo , “Bridging Mice to Men: Using HLA Transgenic Mice to Enhance the Future Prediction and Prevention of Autoimmune Type 1 Diabetes in Humans,” Methods in Molecular Biology 1438 (2016): 137–151. 27150089 10.1007/978-1-4939-3661-8_9

[8] A. K. Mangalam , G. Rajagopalan , V. Taneja , and C. S. David , “HLA Class II Transgenic Mice Mimic Human Inflammatory Diseases,” Advances in Immunology 97 (2008): 65–147. 18501769 10.1016/S0065-2776(08)00002-3

